# Corrigendum: A highly discriminatory RNA strand-specific assay to facilitate analysis of the role of cis-acting elements in foot-and-mouth disease virus replication

**DOI:** 10.1099/jgv.0.001993

**Published:** 2024-05-17

**Authors:** Samuel J. Dobson, Joseph C. Ward, Morgan R. Herod, David J. Rowlands, Nicola J. Stonehouse

**Affiliations:** 1 School of Molecular and Cellular Biology and Astbury Centre for Structural Molecular Biology, Faculty of Biological Sciences, University of Leeds, Leeds LS2 9JT, UK

A reviewer of this article identified that the original published version of this article included an incorrect version of the supplementary data published online in error. The prior supplementary material included averages and standard deviation of pre-analysed raw data which were intended to be removed following peer review.

The corrected supplementary material is available with the online version of this Corrigendum.

The authors express their thanks to the reviewer for identifying this issue and apologise for any inconvenience caused.

## supplementary material

10.1099/jgv.0.001993 Uncited Table S1.

