# Supplementary material for: Corrigendum: A highly discriminatory RNA strand-specific assay to facilitate analysis of the role of cis-acting elements in foot-and-mouth disease virus replication
Source: J Gen Virol. 2024 May 17;105(5):001993. doi: 10.1099/jgv.0.001993 (PMC11256438; doi:10.1099/jgv.0.001993)
Supplement: Uncited Table S1. [file jgv-105-01993-s001.pdf]

Supplementary table 1: Opposite strand challenging does not impact detection of target strand. Stated copies of IVT positive-strand (PS) and negative-strand (NS) RNA were spiked into 500 ng of total RNA extracted from BHK cells. cDNA synthesis and subsequent strand-specific qPCR were performed to determine whether the presence of opposite strand impacted specificity.

Target	PS copies	NS copies	Experiment 1		Experiment 2		Experiment 3	
**PS**	10^5	-	29.62	29.98	30.67	30.11	30.12	30.41
**PS**	10^5	10^5	31.49	32.02	30.1	30.1	29.32	29.5
**NS**	-	10^3	27.19	27.43	26.57	26.73	27.22	27.17
**NS**	10^5	10^3	27.09	27.17	26.84	26.97	27.11	27.34

Supplementary table 2: Raw Cq values of strand-specific qPCR assays described in Figure 1D&E. Reverse transcription reactions were performed using 500 ng total RNA extracted from each condition and positive-strand (PS) specific primer, negative-strand (NS) specific primer or oligo $(dT)_{20}$ (for use with β-actin qPCR primer set). qPCR reactions were performed to detect PS, NS and β-actin (target). N/D = not determined.

Sample	Target	Experiment 1		Experiment 2		Experiment 3	
**tRNA**	**PS**	36.04	36.42	33.28	33.97	34.07	33.87
**tRNA**	**NS**	-	-	-	-	-	-
**tRNA**	**β-actin**	19.92	20	19.68	19.71	20.19	20.16
**GNN**	**PS**	16.33	16.36	15.87	16.08	17.01	17.11
**GNN**	**NS**	26.65	26.76	26.78	26.67	28.06	28.13
**GNN**	**β-actin**	19.84	20.01	19.73	19.83	20.13	20.42
**WT**	**PS**	15.95	16.03	14.95	15.25	15.85	16.07
**WT**	**NS**	25.62	25.73	25.68	25.84	26.81	27.23
**WT**	**β-actin**	20.25	20.37	19.85	20	20.24	20.16

Supplementary table 3: Biotinylation of RNA by click reaction does not significantly impact cDNA synthesis. BHK cells were incubated with/without 0.2 mM 5-EU for 6 hours prior to extraction of RNA. Click reactions were performed to biotinylate labelled RNA and following purification, 500 ng total RNA was reverse transcribed prior to determining the effect of biotinylation upon qPCR assays by detection of β-actin. N/D = not determined.

5-EU	Click	RT	Experiment 1		Experiment 2	
**-**	**-**	-	-	-	-	-
**-**	**-**	+	22.71	22.66	23.14	23.41
**-**	**+**	-	-	-	-	-
**-**	**+**	+	24.07	24.02	23.34	23.32
**+**	**-**	-	-	-	-	-
**+**	**-**	+	22.83	22.89	23.28	23.29
**+**	**+**	-		39.17	-	-
**+**	**+**	+	24.34	24.28	23.44	23.48

Supplementary table 4: Raw Cq values of strand-specific qPCR assays following click reaction and purification of nascent RNA described in Figure 2E&F. BHK cells were transfected with FMDV replicon and incubated with 0.2 mM 5-EU for 6 hours prior to extraction of RNA. RNA was click reacted to label nascent RNA with biotin and purified. Reverse transcription reactions were performed using positive-strand (PS) specific primer, negative-strand (NS) specific primer or oligo $(dT)_{20}$ (for use with β-actin qPCR primer set). qPCR reactions were performed to detect PS, NS and β-actin (target). N/D = not determined.

Sample	Target	Experiment 1		Experiment 2		Experiment 3	
**tRNA**	**PS**	31.50	31.67	30.78	31.69	36.59	37.07
**tRNA**	**NS**	-	-	-	-	-	39.54
**tRNA**	**β-actin**	22.64	22.71	21.83	21.94	27.73	27.53
**GNN**	**PS**	23.95	23.72	23.15	23.19	32.24	31.73
**GNN**	**NS**	35.35	35.77	33.46	34.20	-	39.15
**GNN**	**β-actin**	23.82	24.00	22.59	22.89	29.10	29.22
**WT**	**PS**	18.03	18.01	18.37	18.28	25.77	25.75
**WT**	**NS**	28.83	28.75	29.06	29.12	31.92	31.88
**WT**	**β-actin**	22.79	22.76	23.02	23.19	28.93	29.00

Supplementary table 5: Raw Cq values of strand-specific qPCR assays following click reaction and purification of nascent RNA to determine the effect of A1G and Δ*cre* mutations on strand synthesis described in Figure 3B&C. BHK cells were transfected with FMDV replicon and incubated with 0.2 mM 5-EU for 6 hours prior to extraction of RNA. RNA was click reacted to label nascent RNA with biotin and purified. Reverse transcription reactions were performed using positive-strand (PS) specific primer, negative-strand (NS) specific primer or oligo $(dT)_{20}$ (for use with β-actin qPCR primer set). qPCR reactions were performed to detect PS, NS and β-actin (target). N/D = not determined.

Sample	Target	Experiment 1		Experiment 2		Experiment 3	
**tRNA**	**PS**	32.81	32.35	33.41	33.13	-	-
**tRNA**	**NS**	-	-	-	-	-	-
**tRNA**	**β-actin**	22.48	22.63	23.77	23.84	29.14	28.94
**GNN**	**PS**	24.51	24.75	23.86	24.16	30.09	30.36
**GNN**	**NS**	34.53	38.63	34.98	35.25	36.77	36.86
**GNN**	**β-actin**	23.21	23.29	23.90	23.84	29.67	29.75
**WT**	**PS**	18.99	19.08	18.69	18.93	25.69	26.16
**WT**	**NS**	29.73	29.78	29.15	29.43	31.00	31.62
**WT**	**β-actin**	23.49	23.50	23.31	23.26	29.05	28.51
**A1G**	**PS**	25.01	24.93	22.90	23.12	30.13	30.07
**A1G**	**NS**	35.25	35.73	34.12	33.89	35.60	37.06
**A1G**	**β-actin**	23.44	23.72	23.40	23.52	29.18	29.05
**Δ*cre***	**PS**	24.63	25.00	22.74	22.81	31.60	31.07
**Δ*cre***	**NS**	36.55	-	34.40	34.33	37.87	37.51
**Δ*cre***	**β-actin**	23.22	23.31	22.77	22.62	29.31	29.24
